# New Polyketides from the Antarctic Fungus *Pseudogymnoascus* sp. HSX2#-11

**DOI:** 10.3390/md19030168

**Published:** 2021-03-22

**Authors:** Ting Shi, Yan-Yan Yu, Jia-Jia Dai, Yi-Ting Zhang, Wen-Peng Hu, Li Zheng, Da-Yong Shi

**Affiliations:** 1 State Key Laboratory of Microbial Technology, Institute of Microbial Technology, Shandong University, Qingdao 266200, China; shiting_jia@126.com (T.S.); yuyanyan@sdu.edu.cn (Y.-Y.Y.); daijiajia@sdu.edu.cn (J.-J.D.); 201700140016@sud.edu.cn (Y.-T.Z.); wenpeng19961202@163.com (W.-P.H.); 2 Laboratory for Marine Drugs and Bioproducts of Qingdao National Laboratory for Marine Science and Technology, Qingdao 266071, China; 3 Key Laboratory of Marine Eco-Environmental Science and Technology, First Institute of Oceanography, Ministry of Natural Resources, Qingdao 266061, China; 4 Laboratory for Marine Ecology and Environmental Science, Qingdao Pilot National Laboratory for Marine Science and Technology, Qingdao 266071, China

**Keywords:** Antarctica fungus, *Pseudogymnoascus* sp., polyketides, molecular networking

## Abstract

The species *Pseudogymnoascus* is known as a psychrophilic pathogenic fungus with a ubiquitous distribution in Antarctica. Meanwhile, the study of its secondary metabolites is infrequent. Systematic research of the metabolites of the fungus *Pseudogymnoascus* sp. HSX2#-11, guided by the method of molecular networking, led to the isolation of one novel polyketide, pseudophenone A (**1**), along with six known analogs (**2**–**7**). The structure of the new compound was elucidated by extensive spectroscopic investigation and single-crystal X-ray diffraction. Pseudophenone A (**1**) is a dimer of diphenyl ketone and diphenyl ether, and there is only one analog of **1** to the best of our knowledge. Compounds **1** and **2** exhibited antibacterial activities against a panel of strains. This is the first time to use molecular networking to study the metabolic profiles of Antarctica fungi.

## 1. Introduction

Natural products are the gifts of nature, and have been the major sources of chemical diversity for precursor materials while driving pharmaceutical discovery over the past century [1,2]. Nowadays, the repeating isolation of known compounds has been a tough challenge for natural products research. To resolve this difficulty, Antarctica, with its special climate has been attracting increasing attention. Antarctica, as the southernmost point of the earth, has the most hostile environment, including a cold, dry climate and a low level of nutrition [3]. Microbes, especially fungi, have been proved to have the potential capacity to produce novel compounds to adapt to the extreme habitat. More and more bioactive natural products with novel structures have been isolated from Antarctic fungi [4,5,6,7]. The species *Pseudogymnoascus* is known as a psychrophilic pathogenic fungus with a ubiquitous distribution in Antarctica. However, to the best of our knowledge, there have been only two studies addressing the secondary metabolites of this fungus species to date [8,9].

**Figure 1:**
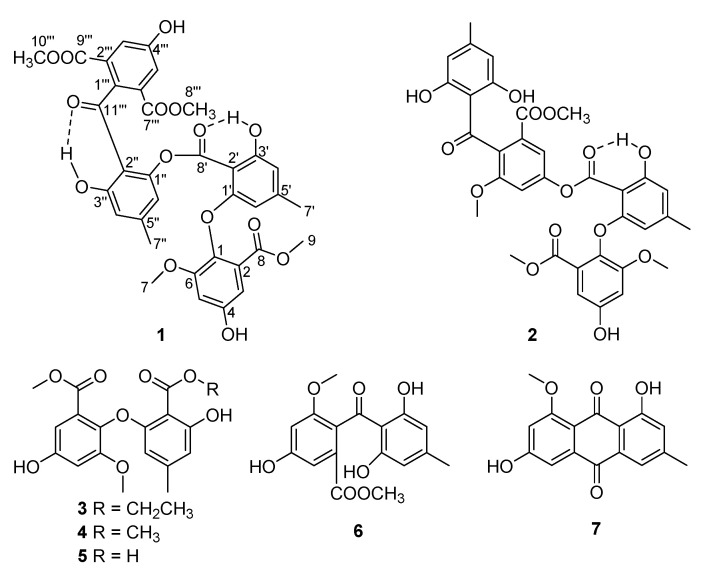
Structures of compounds **1**–**7**.

Molecular networking is an outstanding methodology for time-effective analysis of secondary metabolites in biological samples based on LC-MS/MS fragmentation profiles, and has been proved to be a valuable approach for chemical de-replication and the discovery of new specialized metabolites [10,11,12,13]. Meanwhile, research on the Antarctica fungal metabolic profiles using molecular networking has not been reported. In this research, the secondary metabolic profile of the Antarctic fungus *Pseudogymnoascus* sp. HSX2#-11 was comprehensively studied combining with the method of molecular networking, and led to the isolation of one novel polyketide, pseudophenone A (**1**), along with six known analogs (**2**–**7**) (Figure 1). Pseudophenone A (**1**) has a special structure as a dimer of diphenyl ketone and diphenyl ether. Here, we address the isolation, structure elucidation, and bioactivity evaluation of the isolated compounds.

## 2. Results

### 2.1. Secondary Metabolic Profile Visualization through Molecular Networking

**Figure 2:**
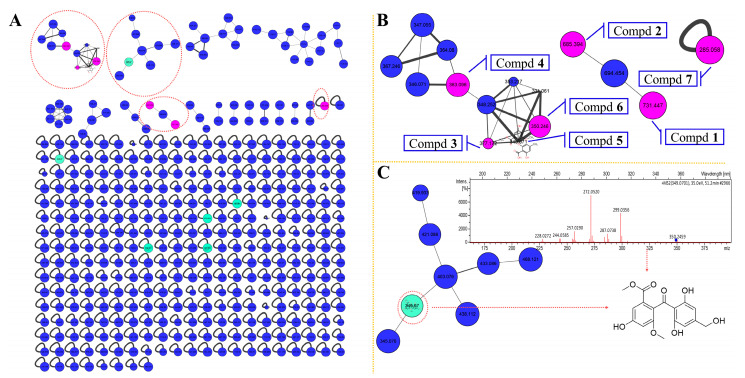
Molecular network of the fungus *Pseudogymnoascus* sp. HSX2#-11. (**A**) Full scan of the secondary metabolites profile of the fungus *Pseudogymnoascus* sp. HSX2#-11 through molecular networking. (**B**) Isolated compounds (purple nodes) in the molecular network. (**C**) The identified, but not obtained, polyketide (green node) in the molecular network.

The fungus *Pseudogymnoascus* sp. HSX2#-11 was cultivated in PDA liquid medium and extracted by EtOAc, MeOH/CH_2_Cl_2_ to obtain organic extracts. Then the extracts were subjected to UHPLC-MS/MS analysis. The afforded MS/MS data were converted into .mzXML format for GNPS searching to get the molecular network (Figure 2), and visualized by Cytoscape. The molecular network of this fungus metabolic profile contained 398 nodes, meaning it had 398 compounds. The green nodes were compounds identified by molecular networking (Table S1). Most of the compounds were unknown after searching the GNPS database visualized through molecular networking. In addition, two identified polyketides which can be clearly seen in two relatively large families of molecular indicated that the fungus *Pseudogymnoascus* sp. HSX2#-11 can produce various polyketides, and the polyketides may be the major producer of this fungus. After isolation of the obtained organic extracts of this fungus, one new polyketide, pseudophenone A (**1**), as well as six known analogs, benzoic acid derivative (**2**) [14], ethyl asterrate (**3**) [15], methyl asterric acid (**4**) [16], asterric acid (**5**) [17], sulochrin (**6**) [17], questin (**7**) [18], were obtained.

### 2.2. Structure Elucidation of Pseudophenone A (***1***)

**Figure 3:**
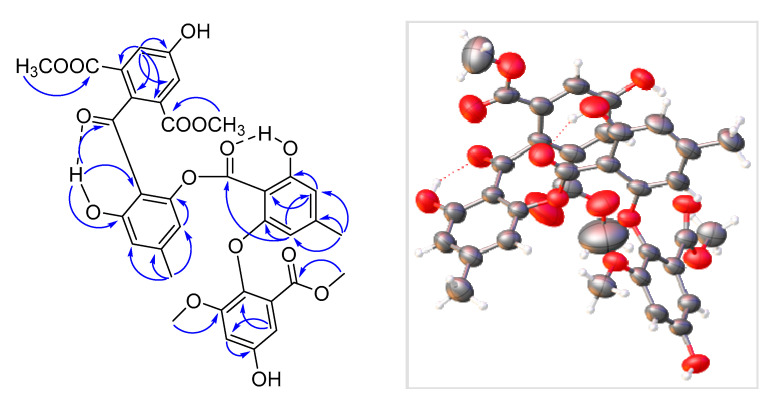
Key HMBC correlations (**left**) and single-crystal X-ray structure (**right**) of pseudophenone A (**1**).

Pseudophenone A (**1**) was obtained as colorless crystals. The molecular formula of C_35_H_30_O_15_ was decided by HRESIMS with the [M + H]^+^ peak at m/z 691.1540 (calcd for C_35_H_31_O_15_, 691.1657) and contained 21 degrees of unsaturation, indicating a highly unsaturated ring system. The ^1^H NMR data of **1** contained eight aromatic proton signals at δ_H_ 6.87 (1H, d, *J* = 2.8 Hz), 6.83 (1H, brs), 6.79 (1H, brs), 6.67 (1H, d, *J* = 2.8 Hz), 6.44 (1H, brs), 6.37 (1H, brs), 6.32 (1H, brs) and 5.87 (1H, brs), combined with the 24 ^13^C NMR signals at 164.1, 162.1, 159.8, 157.4, 154.2, 153.1, 151.1, 147.1, 146.8, 138.2, 125.5, 124.8, 124.8, 124.8, 116.8, 115.1, 113.7, 111.4, 109.1, 108.4, 108.4, 104.8, 103.8 and 101.6, indicating that there were four benzene rings with 16 substituent groups (Table 1). The ^1^H NMR, ^13^C NMR and HSQC spectra (Figures S1–S3) indicated that there were four methoxyls at δ_H_ 3.794 (3H, s), δ_C_ 55.9; δ_H_ 3.791 (3H, s), δ_C_ 52.5; δ_H_ 3.69 (3H, s), δ_C_ 56.2 and δ_H_ 3.64 (3H, s), δ_C_ 52.4, and two methyls at δ_H_ 2.30 (3H, s), δ_C_ 22.1 and δ_H_ 2.15 (3H, s), δ_C_ 22.2. The key HMBC correlations (Figure 3) from H-9 to C-8, H-8‴ to C-7‴ and H-10‴ to C-9‴ established the absence of three substitute groups of –COOCH_3_, while the locations of these substituents were unascertainable. To resolve the question of the structure of **1**, the single crystals of **1** were tried in many solvents. Finally, single crystals of **1** suitable for X-ray diffraction analysis using Cu K*α* radiation were obtained through slow crystallization from the solvent of CDCl_3_ in an environment of 4 °C in a NMR tube (Figure 3). Thus, the structure of **1** was established unambiguously as shown in Figure 1, with a SMILES code of COC(=O)c1cc(O)cc(OC)c1Oc2cc(C)cc(O)c2C(=O)Oc3cc(C)cc(O)c3c5ccc(C(=O)c4c(C(=O)OC)cc(O)cc4C(=O)OC)cc5, and named pseudophenone A. Interestingly, the diphenyl ether portion of **1** was derived from the structures of **3**, **4** or **5**. In addition, the diphenyl ketone part of **1** might be derived from the structures of compound **6**. From the molecular network of the metabolic profile of the fungus *Pseudogymnoascus* sp. HSX2#-11 (Figure 2), there was another polyketide dimer analog with the ions at m/z 694.454.

The structures of **2**–**7** were determined as benzoic acid derivative [13], ethyl asterrate [14], methyl asterric acid [15], asterric acid [16], sulochrin [16], and questin [17], respectively, by comparing their NMR data with those in the literature.

All the isolated compounds (**1**–**7**) were evaluated for their antibacterial activity against a panel of bacteria, including five phytopathogenic bacteria, *X. citri pv. malvacearum*, *X. citri*, *P. syringae*, *D. chrysanthemi* and *E. amylovora*, four animal pathogenic bacteria, *E. coli*, *S. aureus*, *P. aeruginosa* and *B. subtilis*, and eight marine fouling bacteria, *P. fulva*, *A. hydrophila*, *A. salmonicida*, *V. anguillarum*, *V. harveyi*, *P. halotolerans*, *P. angustum and E. cloacae*. Compounds **1** and **2** exhibited antibacterial activities against phytopathogenic bacteria *X. citri pv. malvacearum*, animal pathogenic bacteria *S. aureus*, and marine fouling bacteria *P. fulva* and *A. salmonicida* (Table S4). Compound **1** also showed antibacterial activity against *X. citri* (Table S4).

Compounds (**1**–**7**) were also tested for their cytotoxic activities against five human cancer cell lines A549, HCT116, PANC-1, HepG2 and MDA-MB-231. Meanwhile, none of the isolated compounds showed cytotoxicity.

## 3. Materials and Methods

### 3.1. General Experimental Procedures

UV spectrum was tested through an Implen Gmbh NanoPhotometer N50 Touch (Implen, Germany). NMR spectra were recorded on a Bruker AVANCE NEO (Bruker, Switzerland). Thermo Scientific LTQ Orbitrap XL spectrometer (Thermo Fisher Scientific, Bremen, Germany) was used to measure HRESIMS. UHPLC-MS/MS spectra were tested on a high-resolution Q-TOF mass spectrometry Bruker impactHD (Bruker, Germany), combined with Ultimate3000 UHPLC (Thermo Fisher Scientific, Waltham, MA, USA). The X-ray single crystals were measured by Bruker SMART APEX-II CCD diffractometer (Bruker, Germany). Hitachi Primaide Organizer Semi-HPLC (Hitachi High Technologies, Tokyo, Japan) was performed for HPLC purification. Chromatographic separations were performed using Silica gel (100–200 mesh and 200–300 mesh) and Sephadex LH-20 as stationary phase packing. Thin-layer chromatography was recorded on precoated silica gel GF254 plates.

### 3.2. Fungal Materials

The fungus *Pseudogymnoascus* sp. HSX2#-11 was derived from a soil sample of the Fields Peninsula at Chinese 35th Antarctic expedition in 2019. The strain was deposited in the State Key Laboratory of Microbial Technology, Institute of Microbial Technology, Shandong University, Qingdao, China (NCBI GenBank number: MT367223).

### 3.3. Molecular Networking

#### 3.3.1. UHPLC Parameters

The HPLC C_18_ column (Hitachi, 250 mm × 4.6 mm, 5 µm) was used to perform liquid chromatography. The operating temperature was 30 °C. The UV-detector PDA was measured from 190 to 400 nm for searching compounds, and the detection wavelength of 210 and 254 nm were recorded for characterizing the peaks. The mobile phases were used as MeOH (A) /H_2_O (B). The elution gradient program (time (min), %A) was (0.00, 5); (5.00, 5); (60.00, 100); (75.00, 100); (80.00, 5); (90.00, 5). The injection volume of the sample was 20 µL with 1.00 mL/min flow velocity.

#### 3.3.2. MS/MS Parameters

MS/MS analyses were achieved by high-resolution Q-TOF mass spectrometry using a Bruker impactHD. The ESI source parameters were set as follows: positive-ion mode, capillary source voltage at 3500 V, drying-gas flow rate at 4 L/min, drying-gas temperature at 200 °C, and end plate offset voltage at 500 V. MS scans were recorded in full scan mode with a range of m/z 50−1500 (100 ms scan time) and the mass resolution was 40,000 at m/z 1222.

#### 3.3.3. Molecular Network Analysis

The online workflow (https://ccms-ucsd.github.io/GNPSDocumentation/, accessed on 22 February 2021) was used to form the molecular network on the GNPS website (http://gnps.ucsd.edu, accessed on 22 February 2021) [19]. The UHPLC-MS/MS raw data file was converted into .mzXML format using Bruker Daltonics and deposited at MassIVE with the number of MSV000087079. The MS/MS fragment ions within +/− 17 Da of the precursor m/z were removed to filter the data. The top six fragment ions in the +/− 50 Da window were chosen throughout the spectrum as a window for the filtered MS/MS spectra. The precursor ion mass tolerance was set to 0.1 Da with an ion tolerance of 0.5 Da. The edges were filtered to have a cosine score above 0.7 and more than six matched peaks to create the network. In addition, the edge between two nodes in the network was retained if and only if each node appeared on the other’s top 10 most similar nodes. The maximum size of a molecular family was set to 100, and if the molecular family size was below the threshold, the lowest scoring edges were removed. The spectra in the network were searched in GNPS’s spectral libraries. Each matching between the network spectrum and the library spectrum required a score of 0.7 or more, and no fewer than six matching peaks. The results were visualized using the software package Cytoscape 3.8.0 (Download from https://cytoscape.org/, accessed on 22 February 2021).

### 3.4. Extraction and Isolation

The fungal strain *Pseudogymnoascus* sp. HSX2#-11 was cultivated in a PDA liquid medium in 200 Erlenmeyer flasks (300 mL in each 1000 mL flask) at 16 °C for 45 days. The broth and mycelia were separated through two layers of gauze. Then the mycelia were first extracted by ethyl acetate (EA) three times (3 × 4000 mL) and then with dichloromethane (DCM)/MeOH (MO) (*v*/*v*, 1:1) three times (3 × 4000 mL). The organic extractive broth was obtained through repeated extraction with EA (3 × 60 L). All of the fungal crude extracts were put together and evaporated to dryness under reduced pressure to provide a residue (71.5 g). The residue was subjected to vacuum liquid chromatography (VLC) eluted with EA-petroleum ether (PE) (0–100%) and MO-EA (0–100%) on silica gel to obtain eight fractions (Fr.1–Fr.8). Fr.4 was separated through column chromatography (CC) on Sephadex LH-20 eluted with DCM/MO (*v*/*v*, 1:1) to afford two fractions (Fr.4.1, Fr.4.2). Fr.4.1 was subjected to silica gel CC eluting with EA–PE (0–50%), then purified by using semi-preparative HPLC on an ODS column (Kromasil C_18_, 250 × 10 mm, 5 µm, 2 mL/min) eluted with 65% MO–H_2_O to give compound **3** (2.6 mg). Fr.4.2 was separated on silica gel CC eluted with EA–PE (0–50%) to give compound **7** (3.9 mg). Fr.6 was separated on silica gel Sephadex LH-20 eluted with DCM/MO (*v*/*v*, 1:1) to afford three fractions (Fr.6.1–Fr.6.3). Fr.6.3 was the pure compound **5** (17.6 mg). Fr.6.1 was first eluted with EA–PE (20–100%) on silica gel CC, and then purified through HPLC with 60% and 70% MO–H_2_O for **2** (5.3 mg) and **1** (5.6 mg), respectively. Fr.7 was separated on Sephadex LH-20 CC eluted with DCM/MO (*v*/*v*, 1:1) to get three fractions (Fr.7.1–Fr.7.3). Fr.7.2 and Fr.7.3 were subjected to HPLC with 50% MO–H_2_O to gain **4** (7.0 mg) and **6** (8.7 mg), respectively.

Pseudophenone A (**1**): colorless crystals; UV (CH_2_Cl_2_) λ_max_ (log *ε*): 224 (5.38), 261 (5.10), 325 (4.83); ^1^H and ^13^C NMR data, see Table 1; HRESIMS m/z 690.1583 [M]^+^ (calcd for C_35_H_30_O_15_, 690.1579), [M + H]^+^ m/z 691.1540 (calcd for C_35_H_31_O_15_, 691.1657).

X-ray crystallographic analysis of **1**: C_35_H_30_O_15_, *M*r = 690.59, triclinic crystals, space group P^−1^, *a* = 11.8213(4) Å, *b* = 11.9228(4) Å, *c* = 16.4176(6) Å, *α* = 69.674 (2)°, *β* = 70.338 (2)°, *γ* = 81.306 (2)°, *V* = 2041.80(13) Å^3^, *Z* = 2, *D*_calcd_ = 1.123 mg/cm^3^, *T* = 173 (2) K, *λ* (Cu K*α*) = 1.54184 Å, F(000) = 720, crystal size 0.180 × 0.160 × 0.150 mm^3^, Final *R_1_* value was 0.1135, *wR_2_* = 0.3457 (I > 2σ(I)). Crystallographic data for **1** were given the number CCDC 2064117 after being deposited in the Cambridge Crystallographic Data Centre (CCDC).

### 3.5. Antibacterial Activity Assay

The antibacterial activities were evaluated by the conventional broth dilution assay [20]. Five phytopathogenic bacteria, *Xanthomonas citri pv. malvacearum*, *X. citri*, *Pseudomonas syringae*, *Dickeya chrysanthemi* and *Erwinia amylovora*, four animal pathogenic bacteria, *Escherichia coli*, *Staphylococcus aureus*, *P. aeruginosa* and *Bacillus subtilis*, and eight marine fouling bacteria, *P. fulva*, *Aeromonas hydrophila*, *A. salmonicida*, *Vibrio anguillarum*, *V. harveyi*, *Photobacterium halotolerans*, *P. angustum* and *Enterobacter cloacae*, were used, and cipofloxacin and DMSO were used as positive and negative control, respectively. The initial screening of antibacterial activity assays was tested in 96 well-plate. Each well contained 198 μL bacterial suspension (2–5 × 10^5^ CFU/mL in LB broth) and 2 μL compound (final concentration was 20 μM in DMSO). Three replicates were performed. The plates were incubated at 37 °C for 24 h, then the optic density (OD) values were tested at 600 nm in microplate reader (TriStar^2^ S LB 942 Multimode Reader, Berthold Technologies, Bad Wildbad, Germany). The inhibitory rates were calculated according to the following formula:

Inhibition rate (%) = (OD_DMSO_ − OD_compound_)/OD_DMSO_ × 100

The MIC_50_ values of some active target compounds were evaluated using the 2-fold serial-dilution method. The concentrations of the compounds ranged from 100 µM to 6.25 µM. The other steps were the same as in the primary screening. The MIC_50_ values were calculated using the method of log(inhibitor) vs. normalized response in the software package GraphPad Prism 5.

### 3.6. Cytotoxic Activity Assay

The cytotoxicities against human breast cancer (MDA-MB-231), colorectal cancer (HCT116), lung carcinoma (A549), pancreatic carcinoma (PANC-1) and hepatoma (HepG2) cell lines were evaluated using the SRB method [21]. Adriamycin was used as a positive control.

## 4. Conclusions

In summary, a new polyketide pseudophenone A (**1**), together with six known analogs, were isolated from the Antarctic fungus *Pseudogymnoascus* sp. HSX2#-11, combining with the method of molecular networking. The structure of **1** was determined by extensive spectroscopic investigation and single-crystal X-ray diffraction. Compound **1** is an infrequent dimer of diphenyl ketone and diphenyl ether. The only known similar dimer is **2**, to the best of our knowledge. Another analogous dimer might be contained in the profile of the fungus *Pseudogymnoascus* sp. HSX2#-11 on the basis of the analysis of the molecular network of this fungus. Compounds **1** and **2** exhibited antibacterial activities against phytopathogenic bacteria *X. citri pv. Malvacearum*, animal pathogenic bacteria *S. aureus*, and marine fouling bacteria *P. fulva* and *A. salmonicida* (Table S4). Compound **1** also showed antibacterial activity against *X. citri* (Table S4). Fungi in Antarctica are supposed to produce special compounds to adapt to the extreme environment. The research on Antarctic fungal secondary metabolites is relatively scarcer than on those from other regions, but the research techniques are usually common. This is the first report to use molecular networking to research the secondary metabolic profiles of Antarctic fungi, providing new thinking and methods on investigating novel compounds of Antarctic fungi.

## Figures and Tables

**Table 1:** NMR spectroscopic data (600/150 MHz) for pseudophenone A (**1**) in CDCl_3_.

Position	*δ*_C_	*δ*_H_
1	138.2, C	
2	125.5, C	
3	108.4, CH	6.87, d, (2,8)
4	153.1, C	
5	104.8, CH	6.67, d, (2.8)
6	154.2, C	
7	55.9, CH_3_	3.794, s
8	165.4, C	
9	52.5, CH_3_	3.791, s
1′	159.8, C	
2′	101.6, C	
3′	162.1, C	
4′	108.4, CH	5.87, brs
5′	146.8, C	
6′	111.4, CH	6.37, brs
7′	22.2, CH_3_	2.15, s
8′	168.9, C	
1″	113.7, C	
2″	151.1, C	
3″	115.1, CH	6.32, brs
4″	147.1, C	
5″	116.8, CH	6.79, brs
6″	164.1, C	
7″	22.1, CH_3_	2.30, s
1‴	124.8, C	
2‴	124.8, C	
3‴	109.1, CH	6.83, brs
4‴	157.4, C	
5‴	103.8, CH	6.44, brs
6‴	124.8, C	
7‴	166.0, C	
8‴	52.4, CH_3_	3.64, s
9‴	166.0, C	
10‴	56.2, CH_3_	3.69, s
11‴	199.5, C	
6″-OH		12.75, s

## Data Availability

The fungus *Pseudogymnoascus* sp. HSX2#-11’s ribosomal RNA gene, partial sequence can be found at https://www.ncbi.nlm.nih.gov/nuccore/MT367223.1/, accessed on 22 February 2021; The MS/MS data of the metabolic profiles of *Pseudogymnoascus* sp. HSX2#-11 [doi:10.25345/C53B99] [dataset license: CC0 1.0 Universal (CC0 1.0)] with the number of MassIVE MSV000087079; The detailed crystals data of **1** can be found at https://www.ccdc.cam.ac.uk/structures/Search?Ccdcid=2064117&DatabaseToSearch=Published, accessed on 22 February 2021.

## Supplementary Materials

The following are available online at https://www.mdpi.com/1660-3397/19/3/168/s1, NMR and HRESIMS spectra of **1**: Figures S1–S5; Identified compounds by molecular networking: Table S1; Antibacterial activity assays data: Tables S2–S4.
