# Supplementary material for: New Polyketides from the Antarctic Fungus Pseudogymnoascus sp. HSX2#-11
Source: Mar Drugs. 2021 Mar 22;19(3):168. doi: 10.3390/md19030168 (PMC8004129; doi:10.3390/md19030168)
Supplement: Supplementary file 1 [file marinedrugs-19-00168-s001.pdf]

Supplementary material: New polyketides from the Antarctica fungus *Pseudogymnoascus* sp. HSX2#-11 combing with molecular networking

Ting Shi, Yan-Yan Yu, Jia-Jia Dai, Yi-Ting Zhang, Wen-Peng Hu, Li Zheng, Da-Yong Shi

Figure S1. ^{1}H NMR spectrum of compound **1** ($CDCl_3$).
Figure S2. ^{13}C NMR spectrum of compound **1** ($CDCl_3$).
Figure S3. HSQC spectrum of compound **1** ($CDCl_3$).
Figure S4. HMBC spectrum of compound **1** ($CDCl_3$).
Figure S5. HRESIMS spectrum of compound **1**.
Table S1. Identified compounds by molecular networking.
Table S2. Results of initial antibacterial activity assays.
Table S3. Results of final antibacterial activity assays.
Table S4. MIC_{50} (μM) of compounds **1** and **2**.

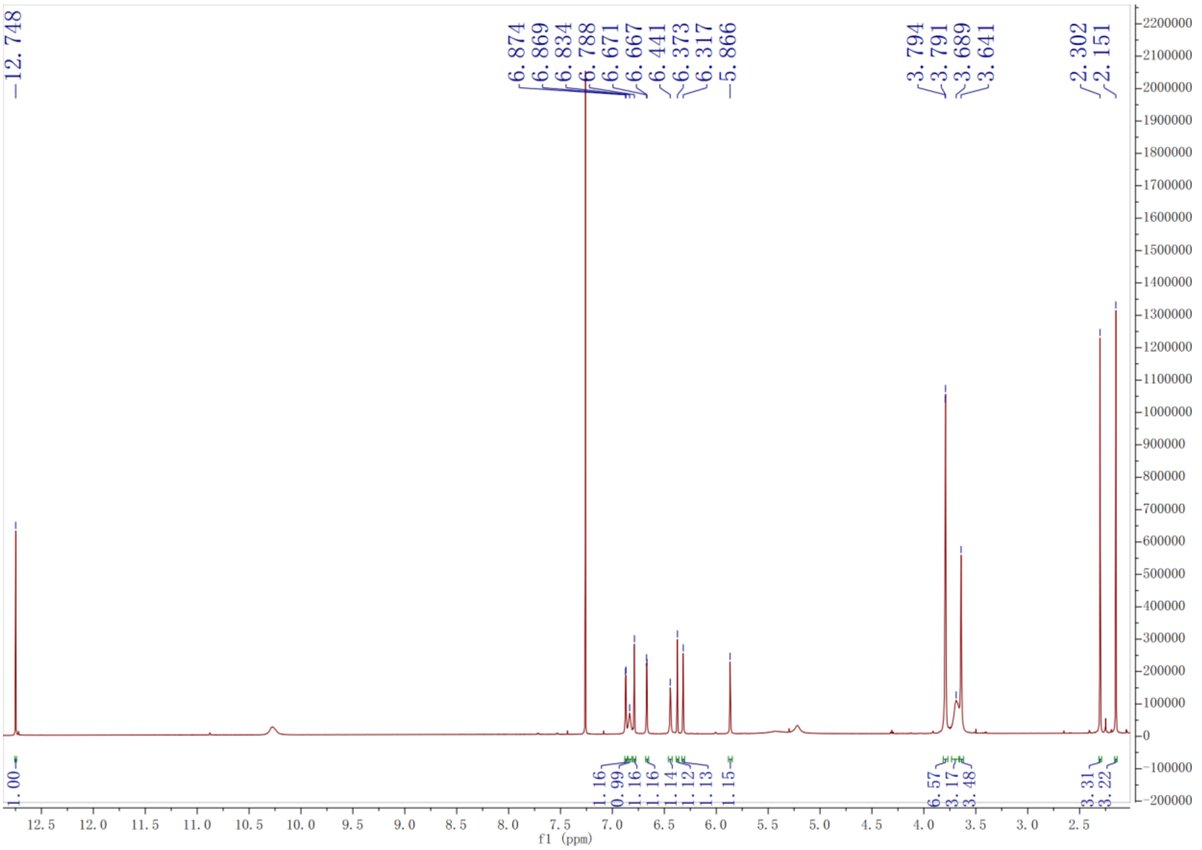

Figure S1. ^{1}H NMR spectrum of compound **1** ($CDCl_3$).

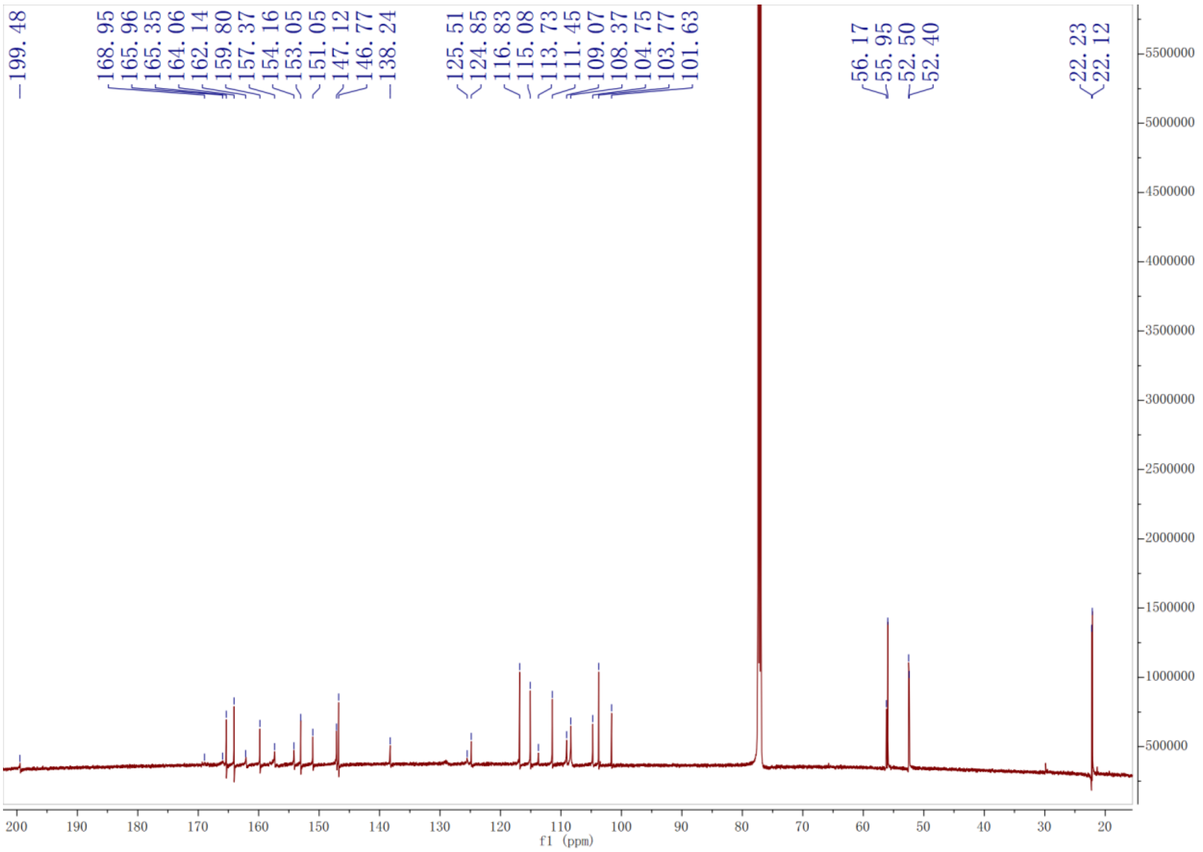

Figure S2. ^{13}C NMR spectrum of compound **1** ($CDCl_3$).

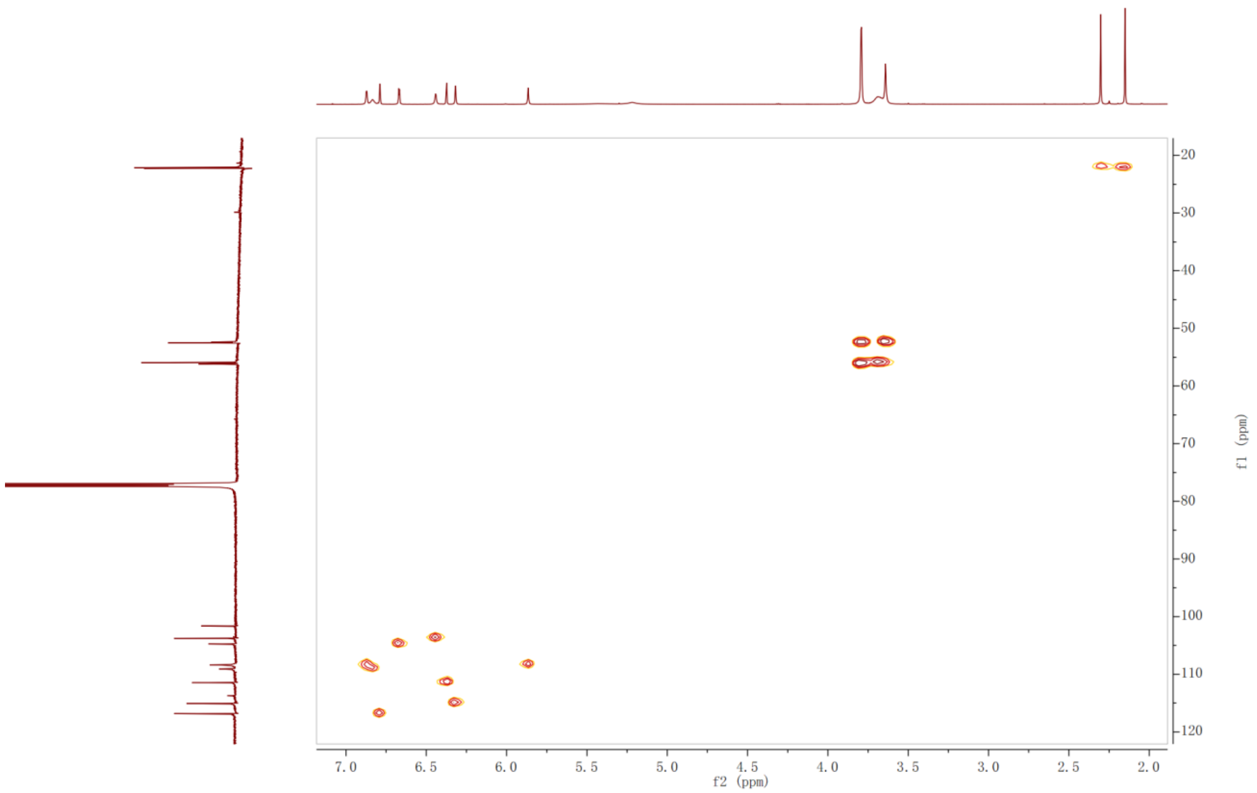

Figure S3. HSQC spectrum of compound **1** ($CDCl_3$).

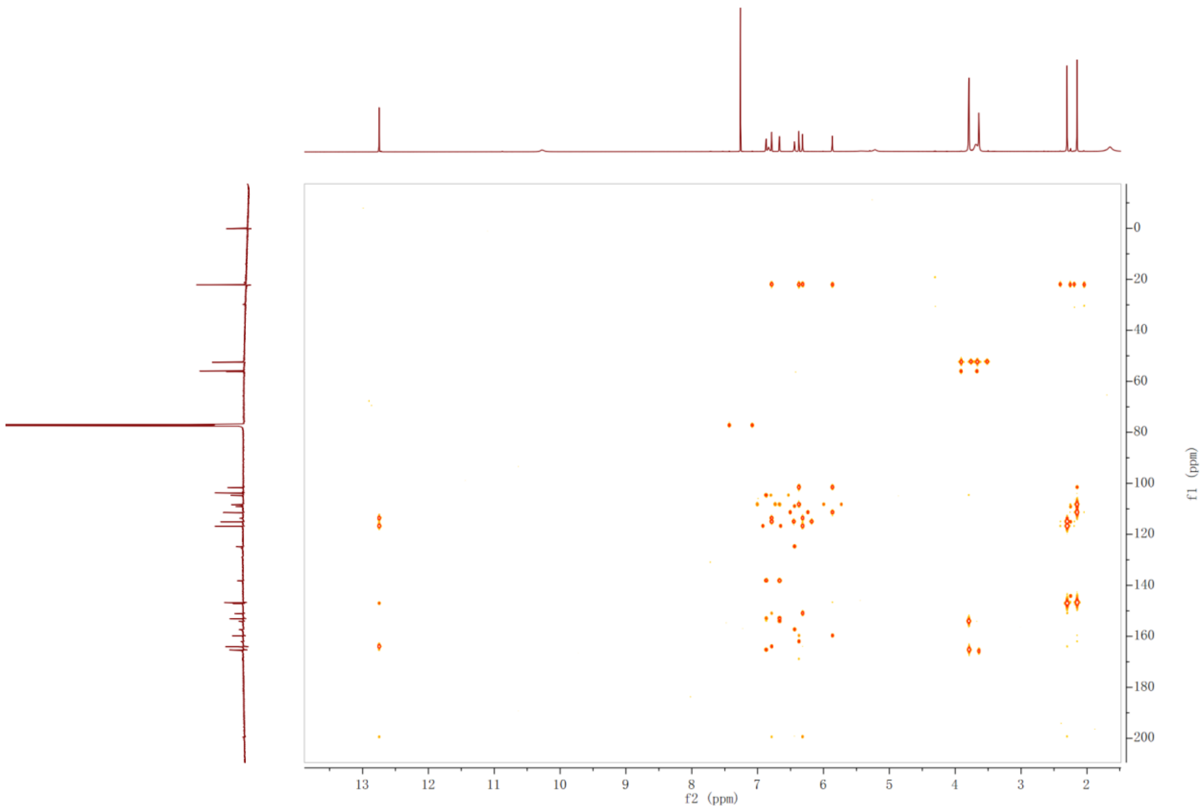

Figure S4. HMBC spectrum of compound **1** ($CDCl_3$).

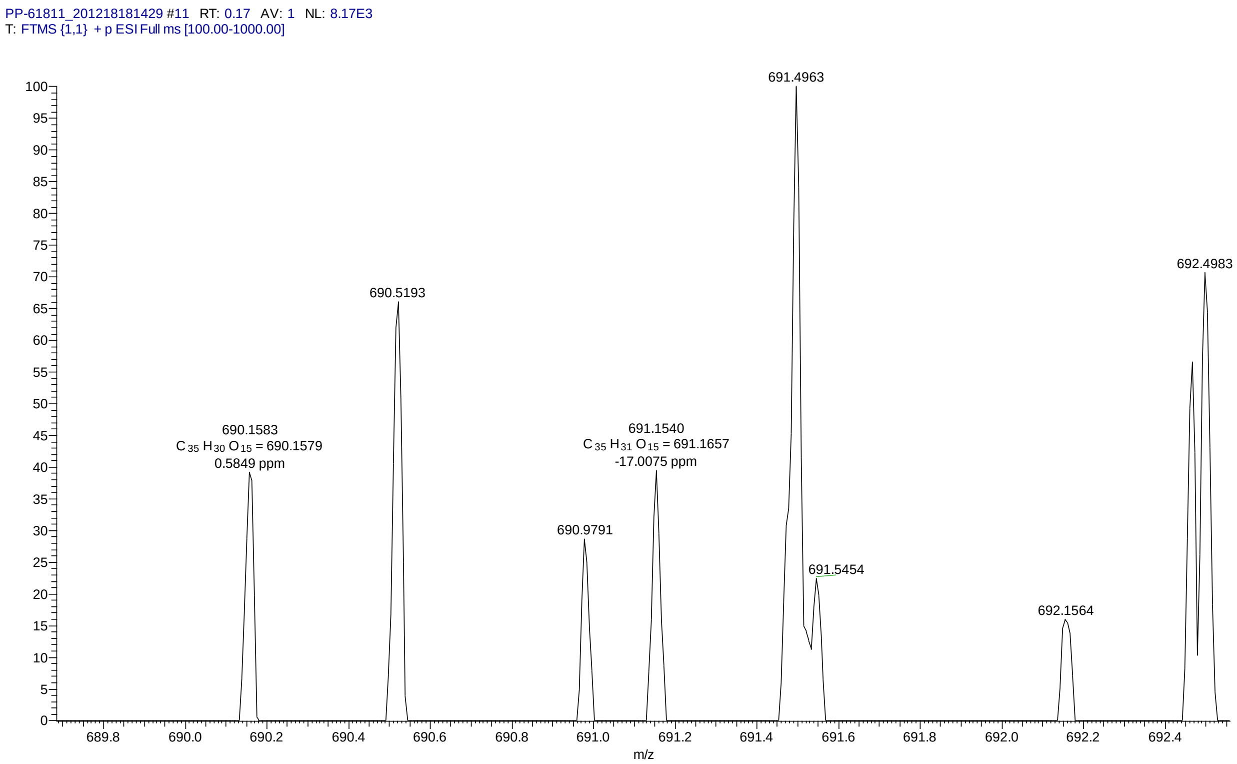

Figure S5. HRESIMS spectrum of compound **1**.

Table S1. Identified compounds by molecular networking

Parent MS	Adduct	Compounds Name
675.633	2M+H	13-Docosenamide
287.061	M+H	Lichexanthone
349.07	M+H	Hydroxysulochrin
349.071	M+H	Asterric acid
807.556	2M+Na	(4*R*)-4-((5*S*,7*R*,9*S*,10*S*,12*S*,13*R*,14*S*,17*R*)-7,12-dihydroxy-10,13-dimethylhexadecahydro-1*H*-cyclopenta [a]phenanthren-17-yl)pentanoic acid
839.541	2M+Na	(2*S*,4*R*)-4-((3*R*,5*S*,7*R*,9*S*,10*S*,13*R*,14*S*,17*R*)-3,7-dihydroxy-10,13-dimethylhexadecahydro-1*H*-cyclopenta [a]phenanthren-17-yl)-2-hydroxypentanoic acid

Table S2. Results of initial antibacterial activity assays.

Compounds	Antibacterial rate (%) [a]							
	**1**	**2**	**3**	**4**	**5**	**6**	**7**	cipofloxacin
X. citri pv. malvacearum	68.96±0.50	42.88±0.55	-5.73±0.25	0.13±1.65	10.62±0.9	19.45±1.41	24.55±0.90	79.74±0.25
X. citri	40.47±0.20	1.06±0.90	-15.87±0.60	-20.00±1.10	-2.52±2.16	4.88±0.98	-1.02±1.54	74.72±2.80
E. amylovora	10.66±0.40	14.04±0.05	12.54±0.75	15.84±0.05	3.15±3.94	0.53±3.44	0.23±0.98	87.08±0.15
P. syringae	-2.13±0.80	4.41±0.35	-24.92±0.80	-31.16±0.55	-7.4±2.36	-14.91±2.52	2.50±1.05	60.67±0.05
D. chrysanthemi	16.59±3.35	-2.51±9.55	14.3±2.15	17.67±2.10	-3.05±1.41	-4.47±1.71	-2.49±2.3	70.86±0.25
S. aureus	55.14±1.15	48.14±0.10	-8.48±0.45	16.84±3.60	-14.97±7.13	-3.85±5.89	-2.10±1.10	83.00±0.40
E. coli	-7.51±3.65	5.50±3.35	-1.42±2.00	-8.59±0.10	-15.97±2.95	-16.22±4.21	-16.99±1.22	81.40±0.35
B. subtilis	1.36±3.10	-5.33±1.80	-29.15±3.10	-26.44±0.30	3.41±4.80	11.09±4.32	7.92±9.40	71.58±0.80
P. aeruginosa	35.95±0.90	13.24±2.75	-42.9±1.30	-41.2±3.15	-7.71±2.38	-4.22±0.75	-7.91±2.00	88.09±0.35

A. salmonicida	78.73±0.40	69.68±0.25	-9.49±0.30	-3.78±1.05	-12.49±2.43	-6.08±3.56	8.14±6.45	79.44±0.35
P. fulva	72.25±2.15	73.03±1.00	11.69±0.20	14.04±1.35	-28.91±6.00	-19.23±3.86	-6.27±3.80	67.06±0.65
A. hydrophila	15.66±1.85	6.49±0.60	-11.22±1.05	-12.97±1.15	4.18±2.10	12.21±6.37	11.17±5.61	88.66±0.10
P. angustum	-3.89±0.45	3.72±1.25	-25.35±0.35	-20.5±0.95	-3.34±2.17	-0.07±1.94	1.34±6.10	78.11±0.35
P. halotolerans	-5.98±0.15	3.13±1.45	-9.81±2.85	-71.54±3.20	0.83±0.46	0.94±7.76	2.54±2.98	80.36±0.35
V. anguillarum	13.07±0.95	20.21±1.10	-7.76±0.25	-47.55±3.90	-9.14±0.66	5.84±13.36	-0.77±12.13	83.15±0.30
V. harveyi	-7.30±1.45	-8.56±0.40	-25.84±0.10	-25.17±2.20	0.46±3.48	3.97±1.25	3.44±1.15	81.71±0.50
E. cloacae	-1.19±1.40	4.10±0.40	-20.9±1.00	-19.25±1.65	2.42±2.14	7.46±3.17	4.20±2.81	85.19±0.40

[a] The initial screening concentration of the compounds **1**–**7** were 20 μM.

Table S3. Results of final antibacterial activity assays.

Comp	Concentration (μM)	*X. citri pv. malvacearum*	*X. citri*	*S. aureus*	*A. salmonicida*	*P. fulva*
**1**	100	76.44±1.04	72.04±0.99	69.93±1.35	82.84±0.57	84.37±2.96
	50	62.66±1.86	66.37±1.93	62.55±1.02	67.95±0.69	62.89±1.07
	25	54.95±0.54	43.1±2.29	48.45±2.25	44.31±1.97	55.58±0.87
	12.5	35.09±0.17	22.66±1.13	23.23±0.93	12.26±3.35	20.88±5.17
	6.25	18.17±0.34	10.4±1.58	-0.99±1.5	14.89±0.77	16.19±2.35
**2**	100	74.19±1.54	-	68.78±4.27	80.99±0.51	86.01±1.67
	50	63.03±4.09	-	56.06±2.83	75.79±1.18	62.05±2.6
	25	55.51±0.92	-	39.55±3.58	46.11±1.28	48.65±3.44
	12.5	20.18±4.05	-	12.23±1.84	35.19±0.48	22.53±7.59
	6.25	15.91±0.97	-	0.15±1.27	19.47±0.33	14.73±1.35

Table S4. MIC_{50} (μM) of compounds **1** and **2**.

Comp	*X. citri pv. malvacearum*	*X. citri*	*S. aureus*	*A. salmonicida*	*P. fulva*
**1**	36.16±4.50	44.19±5.09	47.44±7.21	36.90±2.94	35.64±3.78
**2**	40.83±5.65	-	56.93±6.69	29.86±2.68	37.61±2.99
